# Dengue in Pune city, India (2017–2019): a comprehensive analysis

**DOI:** 10.3389/fpubh.2024.1354510

**Published:** 2024-09-20

**Authors:** Vidya Arankalle, Shubham Shrivastava, Ruta Kulkarni, Rahul Patil, Divya Tiraki, Sanjay Mankar, Rohini Mahesh Taru, Raj Lavange, Arundhati Diwan, Sanjay Lalwani, AkhileshChandra Mishra

**Affiliations:** ^1^Department of Communicable Diseases, Interactive Research School for Health Affairs, Bharati Vidyapeeth (Deemed to be University), Pune, India; ^2^Mankar Children Hospital, Pune, India; ^3^Shivshankar Pote Hospital, Pune Municipal Cooperation, Pune, India; ^4^Om Clinic, Pune, India; ^5^Department of Medicine, Bharati Vidyapeeth (Deemed to be University) Medical College, Pune, Maharashtra, India; ^6^Department of Pediatrics, Bharati Vidyapeeth (Deemed to be University) Medical College, Pune, India

**Keywords:** dengue, symptoms, disease category, primary dengue, secondary dengue, India, DENV

## Abstract

**Objectives:**

To understand the dynamics of dengue disease with special reference to (1) age (2) primary/secondary infections (3) serostatus and (4) serotypes examined during three consecutive years.

**Methods:**

During 3 dengue seasons (2017–19), NS1/IgM ELISAs were used for dengue diagnosis in one of the 15 administrative wards of Pune City, India. Predefined symptoms were recorded at the time of diagnosis/hospitalization. IgG-capture ELISA (Panbio) was used to differentiate primary/secondary infections. DENV serotypes were determined for 260 viral RNA-positive patients.

**Results:**

During the 3 years, 3,014/6,786 (44.4%, 41.4–49.9%) suspected cases were diagnosed as dengue. Use of either NS1 or IgM would have missed 25.5% or 43% of the confirmed dengue cases, respectively. Notably, a higher proportion of secondary dengue cases remained mild while a substantial proportion of primary infections developed warning signs. The symptoms among Dengue/non-dengue patients and primary/secondary infections varied and influenced by age and serostatus. The number and proportion of dengue serotypes varied yearly. A remarkable decline in dengue cases was observed during the COVID-19 pandemic years.

**Conclusion:**

A substantial proportion of primary and secondary dengue patients progress to warning signs/severity or mild infection respectively, underscoring the possible role of non-ADE mechanisms in causing severe dengue that requires hospitalization. Both NS1 and IgM should be used for efficient diagnosis.

## 1 Introduction

Dengue is an ancient arboviral disease caused by dengue virus (DENV) that consists of four distinct serotypes, DENV-1 to DENV-4. Infection with the virus leads to a variety of clinical presentations from subclinical infection and self-recovering mild disease to severe disease with multiple complications needing hospitalization. Fatality is recorded in a variable proportion of the infected patients.

Dengue is an important public health problem in India. The peak season for dengue is the rainy season with increased humidity conducive to generating a large number of mosquito breeding sites. Since the first report of dengue in 1954 (1), the disease has become highly endemic and reported in the urban, rural, and tribal populations and almost all the states across India (2–5). The total number of cases has increased from 101,192 in 2018 to 233,251 in 2022^1^ (6). During this time, no major change in the overall use of diagnostic technologies was witnessed.

Though dengue diagnosis is being provided to an increasing proportion of the Indian population, a substantial proportion of cases, especially the mild ones remain undiagnosed. The use of diagnostic tests (Rapid tests or ELISAs) also depends on the status of the clinical laboratory and location. One of the aims of this study was to estimate the dengue burden employing ELISAs for NS1 and IgM and assess how it compares with the figures provided by the local corporation authorities.

Infection with one serotype does not confer protection against other serotypes and the role of secondary infections in causing severe disease remains largely unknown. In a hyperendemic country like India, an understanding of the association of secondary infection with disease severity and age is essential. Antibody-dependent enhancement (ADE) caused by the heterotypic infection in a previously exposed individual is an important phenomenon in dengue. The implication of ADE in enhancing disease severity remains a matter of concern (7). Taken together, longitudinal data on the association of primary/secondary infections and disease severity is critical, especially when a well-planned serosurvey in Pune documented that over 81% of individuals were positive for DENV antibodies (8).

When the present study was undertaken in 2017, information on the prevalent serotypes over time was unavailable from India. Subsequently, studies during the same time have been reported (9–14). In two states in South India (9, 10), the patterns were different. In central India, both in 2019 and 2021, all 4 serotypes were detected, DENV-2 being predominant (11). Two studies from the same state but at different locations in North India reported distinct differences in the circulating serotypes determined during different years (12, 13). In Delhi, DENV-2 was the predominant serotype during 2011–2015 that was replaced by DENV-3 during 2016–2017 (14). Clearly, differential serotype patterns were recognized in different parts of the country and, within the same region.

Pune City with a population of 94,29,408^2^ experienced sporadic cases in the 1970s and 80s. In the 90s, seasonal outbreaks were recorded in different localities of the city (15). Notably, haemorrhagic involvement was documented in some cases. Though dengue appears yearly, the disease burden is not known. Despite being a reportable disease (16), a multiplication factor of 200 is suggested by using various models for a close-to-true estimate (17, 18). Administratively, Pune City is classified into 5 zones that are divided into 15 administrative units called wards. For this study, we selected one ward, Dhankawadi that reports dengue outbreaks yearly and a seroprevalence of 75% in 2017 (8).

In the absence of comprehensive data on disease burden, severity of primary/secondary infections in children and adults, and virus serotypes we decided to generate such data for three consecutive years (2017–2019). Such information is also crucial in evaluating the efficacy of dengue vaccines in clinical trials and real-world settings as soon as the vaccines are approved for human use.

## 2 Methods

### 2.1 Ethics statement and data collection

This study was approved by the Institutional Human Ethics Committee (IEC/2017/04, IEC/2018/11, IEC/2019/06). Blood samples were collected only after informed consent from the patients or parents/guardians of the paediatric patients. Symptoms at the time of blood collection and other relevant information were collected using a preset proforma. For this, tablets were used, and the data was electronically transmitted to the laboratory.

### 2.2 Study area

For dengue surveillance in Pune city, Dhankawadi ward was selected as dengue cases are reported every year. Vicinity to the institute was an additional advantage. Within the ward, one municipal corporation hospital, one tertiary care private hospital (Bharati hospital), and 10 general practitioners (GP) catering to different parts of the ward were selected. As the proportion of children was low, one paediatric hospital from an adjacent ward was added in the second year. The blood samples for dengue diagnosis and required information were collected from June to December in all 3 years. Sample collection at admission was done daily every morning for all the hospitals whereas patients attending both morning and evening GP clinics were included. A patient with fever and two or more of the following: (nausea/vomiting, rash, aches and pains, tourniquet test positive, leukopenia) and any warning signs was classified as a suspected dengue case. Suspected cases positive for DENV NS1 or IgM-anti-DENV or both were diagnosed as dengue. For the confirmed dengue cases (*n* = 3014), the time between the onset of illness and blood collection was a median of 4 days (IQR = 3–6 days). No follow-up samples or information was collected. A retrospective analysis of case records was done for the dengue patients admitted to Bharati hospital (*n* = 453). This included daily clinical/biochemical follow-up till discharge/death.

### 2.3 Serology

Serum samples from all the suspected dengue patients were immediately tested for DENV NS1 antigen (Dengue NS1 Ag MICROLISA, J. Mitra and Co. Pvt. Ltd., New Delhi, India) and anti-DENV IgM antibodies (Panbio Dengue IgM Capture ELISA, Alere Inc., Australia). The remaining samples were stored at −70°C in aliquots till tested. The patients positive for either NS1 or IgM or both markers were diagnosed as dengue. To distinguish between primary and secondary infections, samples from serologically confirmed dengue patients were further screened for anti-DENV IgG antibodies using Panbio Dengue IgG Capture ELISA (Panbio; Alere Inc., Australia). According to this ELISA, the detection of IgG-antibodies at a concentration > 22 Panbio units (equivalent to haemagglutination inhibition titer of 2,560) is considered positive for secondary dengue infection. Due to limited sample volumes, this test could not be done in all dengue-positive samples.

### 2.4 DENV serotyping

Total RNA was extracted from 140 μL of human serum using a QIAmp viral RNA kit (QIAGEN, INC, Valencia, CA), as per manufacturer’s protocol. RNA was eluted in 50 μL of AVE buffer provided with the kit. For serotyping, CprM region of the viral genome was amplified as reported earlier (19). CprM sequences were confirmed by BLAST analysis^3^ to confirm the serotypes.

### 2.5 Statistical analysis

Epi-Info software (Version- 1.6, CDC, https://www.cdc.gov/epiinfo/index.html) was used to collect clinical information on all the suspected dengue patients visiting selected clinics and hospitals. This information was gathered in one location. The proportion test was used to find the statistical significance between two proportions of categorical data. Different table statistics were obtained to summarize the symptoms data; median and IQR were defined for age groups. Microsoft Excel 365 and Power-BI (Version- 2.121.762.0 64-bit) were used to handle and maintain data for different years separately. As per the study requirement, we merged and appended different data frames, and used Excel pivot tables to summarize the data. For graphical representation, Graph Pad Prism (Version - 10.2.0) was employed. R software (Version 4.3.1) was used to analyse statistical significance using the hypothesis testing concept. To create new variables from existing ones, the concept of data engineering was used whenever needed. *p*-value <0.05 was considered significant.

## 3 Results

**Figure 1:**
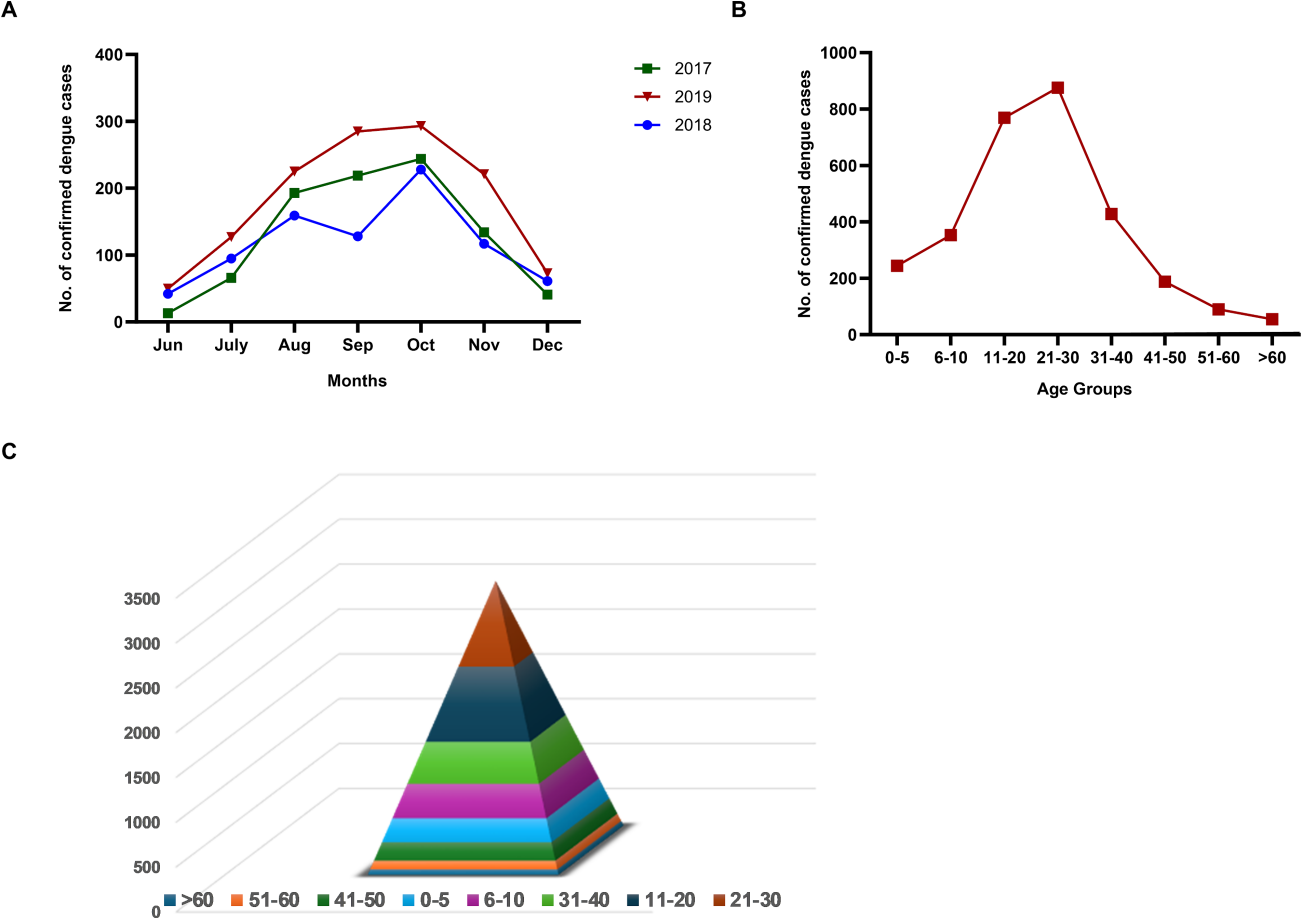
**(A)** Month-wise number of dengue cases during three consecutive years (2017–2019), **(B)** Age-wise distribution of dengue cases diagnosed during 2017–19, and **(C)** Age group-wise dengue cases in a pyramid structure.

The study was conducted for 3 years (2017–2019). To capture all the dengue cases in a season, the serologic testing was initiated in June and continued till December. During the study period, 3014/6786 (44.4%) suspected cases were serologically confirmed as dengue. Month-wise distribution of dengue cases during the three seasons exhibited a similar pattern (Figure 1A). The rise in the number of cases commenced in July, increased during August–October, and started declining in November. The age distribution of dengue patients showed that the 21–30 years age group was maximally affected followed by 11–20 years (Figure 1B). A steep rise in the cases among >10 years of age was noteworthy. The proportion of patients in the 40+ age group was low. Figure 1C depicts age-wise dengue cases as a pyramid.

### 3.1 Patient characteristics

Table 1 provides details of the dengue patients diagnosed during the three dengue seasons investigated. Of the 6786 suspected cases, 44.4% (varied from 40.8 to 49.9% in 3 years) were serologically confirmed as Dengue. Males were affected 1.5 times (1.4–1.6) more than females with the predominance of adults (63.3%, 61.1–69.1%). The median ages among children and adults were 10 (IQR 6–13) and 28 (IQR 22–36) years, respectively. Out of 3014 confirmed dengue cases, 2,476 cases (82.1%) could be categorized further as primary and secondary dengue infections mainly due to inadequate quantity and use in other tests. Primary and secondary dengue could be classified for 81.9% of the children (881/1,060) and 82.2% (1,595/1,939) adults. Throughout the study period of 3 years, primary infections (*n* = 1,561, 63.1%) were higher than the secondary (*n* = 915, 36.9%; *p* < 0.001). A similar trend was seen for both age groups (*p* < 0.001). Each year, there was no difference in the proportions of primary and secondary infections in both age groups (*p* = 0.07–0.4). The difference in primary infections in children investigated in 2017 and 2018 (*p* = 0.03) was most likely due to the addition of the paediatric hospital in 2018 that continued in 2019. The overall median (IQR) ages in primary and secondary dengue among children and adults were 10 (6–13), 11 (7–14) and, 27 (22–35), 29 (23–38) years, respectively.

**Table 1:** Characteristics of the dengue patients diagnosed during the 2017–19 dengue seasons (Jun–Dec).

Dengue cases	2017	2018	2019	Total
Suspected	2199	2032	2555	6786
Confirmed	910 (41.4%)	830 (40.8%)	1274 (49.9%)	3014 (44.4%)
M: F ratio	1.5: 1	1.6: 1	1.4: 1	1.5: 1
<18 Years of age	281 (30.9%)	298 (35.9%)	496 (38.9%)	1075 (35.7%)
Median age year (IQR)^#^	11 (6–14)	10 (6–13)	10 (6–13)	10 (6–13)
≥18 Years of age	629 (69.1%)	532 (64.1%)	778 (61.1%)	1939 (63.3%)
Median age year (IQR)	28 (23–35)	29 (22–39)	28 (22–36)	28 (22–36)
Age-wise primary and secondary dengue infections*				
<18 Years				
Primary No (%)	174 (71.7)	201 (67.9%)	247 (73)	622 (70.1%)
Median age, year (IQR)	11 (7–14)	10 (6–13)	9 (5–13)	10 (6–13)
Secondary No (%)	69 (28.3)	95 (32.1%)	95 (27)	259 (29.4%)
Median age, year (IQR)	12 (9–14)	11 (7–15)	9 (6–13)	11 (7–14)
≥18 Years				
Primary No (%)	378 (61.4)	269 (56.2%)	292 (59.5)	939 (59%)
Median age, Year (IQR)	27 (22–34)	28 (22–37)	28 (22–35)	27 (22–35)
Secondary No (%)	238 (38.6)	213 (43.8)	205 (40.5)	656 (41%)
Median age, year (IQR)	28 (23–36)	30 (23–40)	30 (24–37)	29 (23–38)
Total dengue cases				
Primary + Secondary	859	778	839	2476
Primary No (%)	552 (64.3%)	470 (60.4%)	539 (64.2%)	1561(63.1%)
Median age, year (IQR)	22 (13–30)	16 (10–28)	20 (13–32)	22 (13–30)
Secondary No (%)	307 (35.7%)	308 (39.6%)	300 (35.8%)	915 (36.9%)
Median (IQR)	26 (18–33.25)	23 (14–31)	23 (15–35)	25 (17–34)

*Due to insufficient quantity, sera from 81.9% of the children and 82.2% of adults were tested for primary/secondary dengue classification. ^#^IQR (Inter-quartile range showing Q1–Q3 values).

### 3.2 Comparison of symptoms presented by dengue and non-dengue patients identified during the study

**Figure 2:**
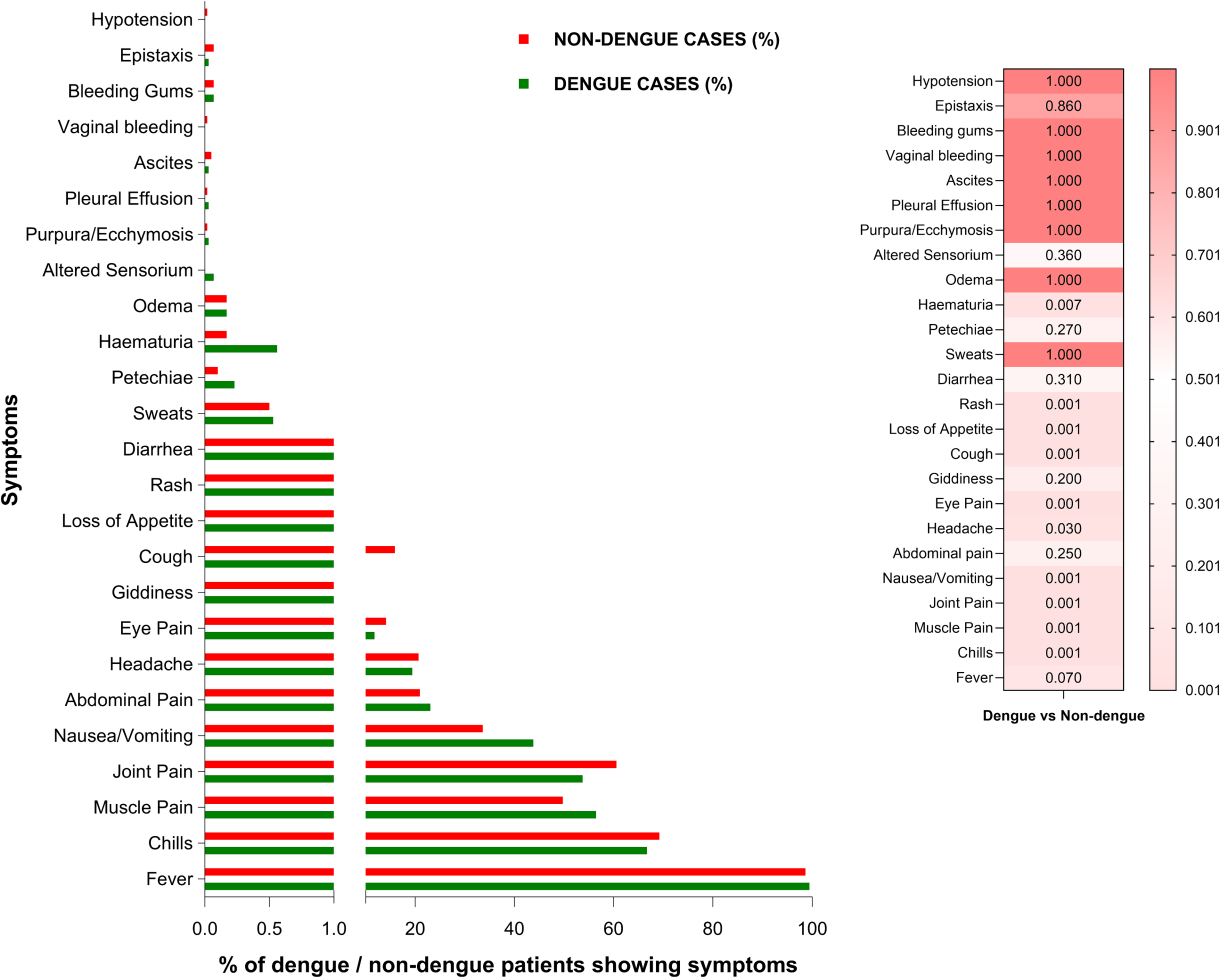
Comparison of clinical symptoms exhibited by dengue (*n* = 3,014) and non-dengue (*n* = 3,772) cases representing three dengue seasons. Heatmap depicts *p*-values for the comparison of specific symptoms in the two groups using a proportion test. *p* < 0.05 is considered significant.

Figure 2 depicts comparisons of symptoms presented by dengue (*n* = 3,014) and non-dengue (*n* = 3,772) patients studied during 2017–19. The frequency of loss of appetite, nausea/vomiting, muscle pain, rash (*p* < 0.001 for all), and hematuria (*p* = 0.02) was higher in dengue patients while chills, joint pain, eye pain, cough (*p* < 0.001 for all) and headache (*p* = 0.03) were reported more frequently by the non-dengue patients. We did not attempt to provide a specific diagnosis for non-dengue patients.

### 3.3 Relationship of NS1/IgM seromarkers with the duration of symptoms, type of infection, and clinical presentation

During dengue infection, NS1 is the earliest seromarker followed by the appearance of IgM antibodies. Subsequently, NS1 disappears while IgM persists for a variable period. The presence of NS1 denotes the viraemic phase, NS1 alone positive patients are likely to be more viraemic and with high viremia levels than those with IgM antibodies (20).

**Figure 3:**
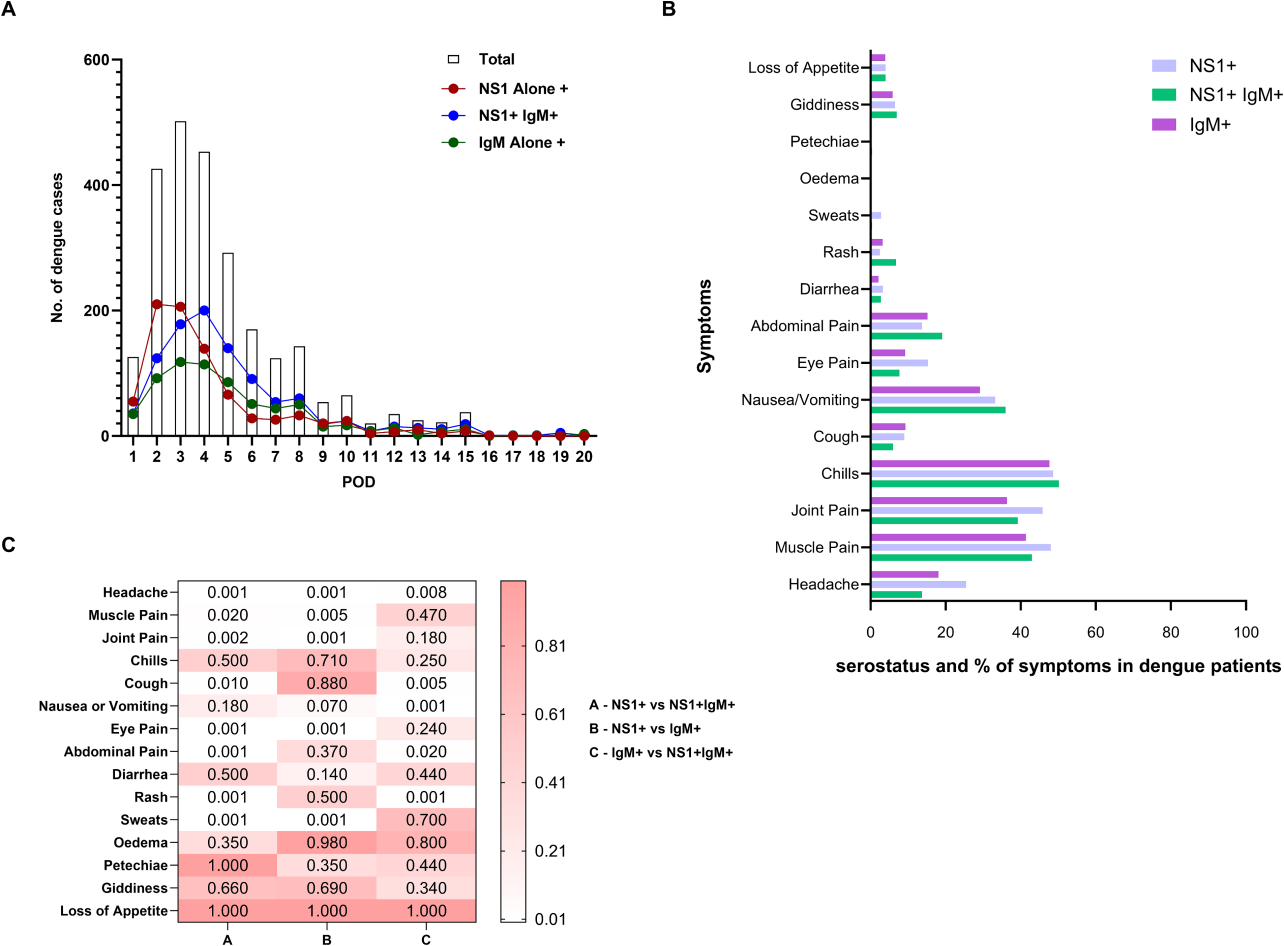
**(A)** Depicts the relationship of post-onset-day of clinical symptoms at the time of collection with the total number of dengue cases (clear column), NS1 alone positive patients (NS1+, red circle), patients positive for both the markers (NS1+ IgM+, blue circle) and anti-DENV-IgM alone positive patients (IgM+, green circle). **(B)** Comparison of proportion of symptoms among patients with NS1 alone, NS1 + IgM+, and IgM alone seromarkers. **(C)** Heatmap depicts the *p*-value significance between the groups showing clinical symptoms (group A - NS1+ vs. NS1 + IgM+), (group B – NS1+ vs. IgM+) and (group C – IgM+ vs. NS1 + IgM+). *p*-values were calculated using the “proportion test” and *p*-value <0.05 was considered significant.

Figure 3A illustrates the dynamics of NS1/IgM seromakers for 20 days post-onset of clinical symptoms. NS1 alone positivity peaked on Post-onset Day 2 (POD-2) while NS1 + IgM+ and IgM alone reactivity peaked on days 4 and 3, respectively. Importantly, NS1 positivity with or without IgM declined sharply while IgM decline was slower. Of the 2,606 patients tested for NS1 and IgM, 71.1, 22.7, and 6.2%, respectively, visited healthcare facilities within 5 days, 6–10 days, and 11–20 days post-onset of clinical symptoms. Within 5 and 6–10 days, overall NS1 positivity was 75.4% (38% without IgM and 37.4% with IgM) and 68.6% (26% without IgM and 64% with IgM) respectively. During 11–20 POD, NS1 positivity was 68.5% (23.4% without IgM and 45.1% with IgM). Taken together, if only NS1 or IgM were used for diagnosis, we would have diagnosed only 74.5% or 57% of the confirmed dengue cases, respectively. Clearly, in real-world settings, both NS1 and IgM testing is necessary to avoid gross underestimation of the disease.

Next, we compared the proportions of NS1/IgM positivity with primary/secondary dengue infections. Irrespective of age, NS1 alone-positive patients presented mainly with primary infection (283/300, 94.3% in children and 504/543, 92.8% in adults, *p* < 0.001 for both). Among the paediatric primary dengue patients, NS1 + IgM+ positivity was also predominant (316/433, 73%, *p* < 0.001) while adult secondary dengue patients exhibited higher IgM alone positivity (361/499, 72.3%, *p* < 0.001). Taken together, NS1 positivity with or without IgM was most predominant among primary infections while IgM alone positivity mainly indicated secondary infections.

### 3.4 NS1 alone positives are more symptomatic

Our next aim was to compare pre-defined symptoms among dengue patients classified based on serostatus, primary/secondary disease, and age. To assess the relationship between symptoms and NS1/IgM serologic phases that reflect disease duration, the proportions of different symptoms were compared as per the status of the seromarkers (Figures 3B,C). Higher proportions of NS1 alone positive patients reported headache, muscle pain, joint pain, eye pain, and sweats (*p* = 0.005- < 0.001) than those positive for NS1 and IgM while abdominal pain and rash (*p* < 0.001 for both) were reported more frequently by the patients positive for both the markers. Overall, NS1 + IgM- patients (early acute phase) were more symptomatic than those with both the markers and IgM-alone positive patients (late phase).

### 3.5 Adults with primary and secondary infections are more symptomatic than the respective paediatric patients

**Figure 4:**
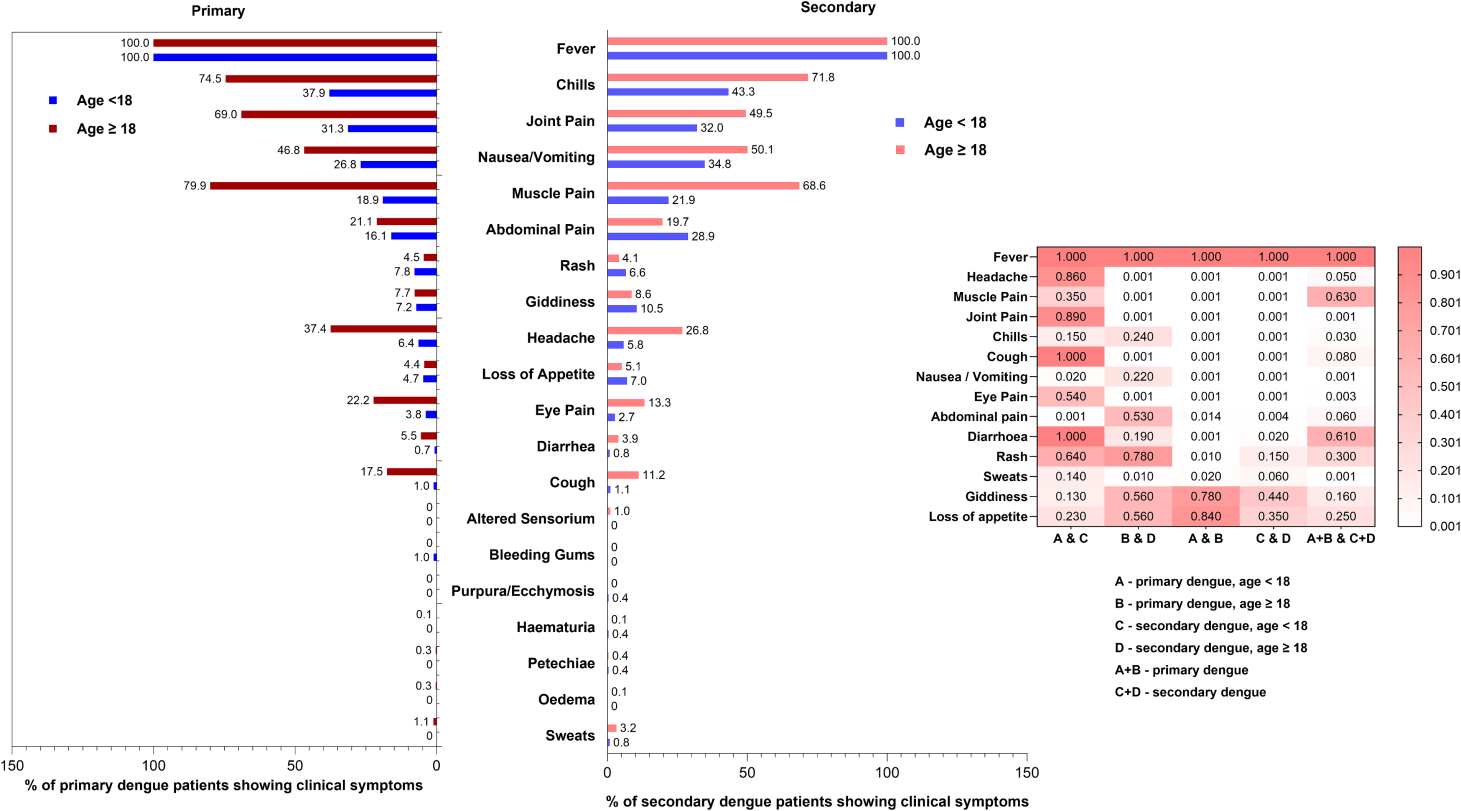
Age-wise comparisons of proportion of symptoms exhibited by primary and secondary dengue cases. Heatmap depicts differences among (1) primary and secondary infections among paediatric and adult dengue patients (2) paediatric as well as adult primary dengue patients and (3) total primary and secondary dengue patients. The *p*-values were determined using the “proportion test.” *p*-value of <0.05 was considered significant.

Next, we attempted to understand age-wise differences in the proportion of primary and secondary dengue patients presenting with different symptoms at the time of first visit to clinics or hospital admission (Figure 4). Of the 25 symptoms included in the case history form, ascites, epistaxis, pleural effusion, hypotension, and vaginal bleeding were not reported at the time of recruitment and first blood sample collection. Subsequent follow-up was not undertaken. Fever was present in all the patients examined. With the limitation of the possibility of the inability of younger children or their parents to express specific symptoms, we found that abdominal pain (*p* = 0.001) and nausea/vomiting (*p* = 0.02) were more common in secondary dengue among children. During primary infections, the rash was more frequently seen in children (51/652, 7.8%) than the adults (41/908, 4.5%, *p* = 0.01). Of note, 17.9% of 1132 children included were < 5 years and the remaining should have been able to describe the symptoms. A higher proportion of adult, primary dengue patients reported headache, muscle pain, joint pain, cough, and eye pain (*p* = 0.01- < 0.001). No difference was recorded among adult primary and secondary dengue.

### 3.6 Relationship of age, warning signs, and primary/secondary dengue infections

As disease severity is shown to be associated with the age of patients and the type of infection, i.e., primary or secondary, we compared these variables. For determining disease severity, the WHO-2009 classification was used: patients (1) without warning signs (WWS, *n* = 2042), (2) with warning signs (WS, *n* = 571), and (3) severe disease (SD, *n* = 3). As the number of SD cases was only 3, they were clubbed with the WS group for all further analyses.

### 3.7 Age and proportion of patients with warning signs

Table 2 depicts the overall association of age with warning signs. The proportion of patients with warning signs was independent of age (*p* = 0.15) whereas WWS patients were higher in the adult category (*p* < 0.001). The maximum WS cases among children and adults were 5–12 (median 8) and 20–37 (median 27) years of age.

**Table 2:** Age-wise proportion of patients with or without warning signs.

		2017–19: Age-wise No./Total cases (%)			
Category		WS (Gr A) median age (IQR)	WWS (Gr B) median age (IQR)	p-value (A&B)	No. (SD)
Children (Gr C)	Positive cases/Total cases (%)	272/569 (47.8%)	678/2045 (33.1%)	<0.001	0
	Median Age (IQR)	9 (6–13)	10 (6–13)	0.27	
Adults (Gr D)	Positive cases/Total cases (%)	297/ 569 (52.2%)	1367/2045 (66.9%)	<0.001	3
	Median Age (IQR)	29 (23–36)	28 (22–36)	0.66	
*p*-value (C&D)	Positive cases/Total cases (%)	0.15	<0.001		

### 3.8 Warning signs, age, and primary/secondary infections

Next, we assessed age-wise proportions of patients with warning signs among primary and secondary infections. WS and WWS children exhibited a higher proportion of primary infections during all 3 years (*p* < 0.001 for all, Table 3). In the WWS group, primary infections were higher than secondary (*p* < 0.001 for all, both groups). However, in the adults with WS, secondary infections were higher in 2017 and 2019 (p < 0.001) while in 2018, there was no difference between primary and secondary infections (*p* = 0.8). Overall, secondary infections were higher in adults with warning signs. When the median age groups across the study and each year were compared, both in WS and WWS categories, the median age was always higher in the secondary than that of primary dengue infection (*p* = 0.002 - <0.001).

**Table 3:** Proportion of patients with or without warning signs in primary/secondary dengue infections.

Study year, age in years	Patients with warning signs (WS)			Patients without warning signs (WWS)		
	Primary no (%)	Secondary no (%)	*p*-value	Primary no (%)	Secondary no (%)	*p*-value
2017, all ages	87 (62.1)	53 (37.8%)	<0.001	462 (64.9%)	250 (35.1%)	<0.001
Median (IQR)	17 (10–25)	22 (14–28)	<0.001	24 (15–31)	27 (19–35)	<0.001
<18 years	46 (52.9)	20 (37.7)	<0.001	144 (31.2)	54 (21.6)	<0.001
Median (IQR)	10 (5.25–13)	10 (8–14)	0.14	10.5 (6–14)	12 (9–14)	0.35
> = 18 years	41 (47.1)	33 (62.3)	<0.001	318 (68.8)	196 (78.4)	<0.001
Median (IQR)	25 (21–30)	27 (23–35.5)	0.52	27 (23–35)	28 (24–37)	0.82
2018, all ages	89 (55.5)	71 (44.4%)	0.06	486 (63.8%)	276 (36.2%)	<0.001
Median (IQR)	21 (10–32)	26 (17–34)	<0.001	20 (12–29)	23 (15–33)	<0.001
<18 years	36 (40.4)	21 (29.5)	<0.001	205 (42.2)	81 (29.3)	<0.001
Median (IQR)	9 (7–11.5)	12 (10–16)	0.56	10 (6–14)	10 (6–12)	0.25
> = 18 years	53 (59.5)	50 (70.4)	0.8	281 (57.8)	195 (70.6)	<0.001
Median (IQR)	30 (24–36)	30 (24–41)	0.47	28 (22–37)	30 (22–40)	0.35
2019, all ages	168 (62.4)	101(37.5%)	<0.001	371 (65.0%)	200 (35.0%)	<0.001
Median (IQR)	15 (8–26)	16 (8–29)	<0.001	20 (10–29)	27 (18–35)	<0.001
<18 years	95 (56.5)	54 (53.5)	<0.001	152 (41)	42 (21)	<0.001
Median (IQR)	9 (6–12)	8.5 (7–12)	0.01	9 (5–13)	10 (5–14.5)	0.37
> = 18 years	73 (43.4)	47 (46.5)	<0.001	219 (59)	158 (79)	<0.001
Median (IQR)	29 (22–36)	30 (24.5–38)	0.72	27 (22–35)	30 (23–37)	0.75
Total	344 (60.4%)	225 (39.6%)	<0.001	1319 (64.5%)	726 (35.5%)	<0.001
Median (IQR)	17 (9–28)	21 (11–30)	0.002	21 (12–30)	26 (18–35)	<0.001
<18 years	177 (51.4)	95 (42.2)	<0.001	501 (38)	177 (24.4)	<0.001
Median (IQR)	8 (5–11)	9 (7–11)	0.07	8 (5–12)	9 (6–12)	0.006
> = 18 years	167 (48.5)	130 (57.8)	<0.001	818 (62)	549 (75.6)	<0.001
Median (IQR)	26 (20–34)	28 (22–36)	0.13	26 (21–34)	28 (22–37)	0.051

### 3.9 Retrospective analysis of the patients admitted to Bharati hospital

A separate retrospective analysis was done for 453 patients admitted to Bharati hospital during the study duration. Clinical and biochemical parameters advised by the treating clinicians were available till discharge/outcome. With this analysis, WS cases increased from 102 to 281, the number of patients with severe disease from 3 to 14, including one fatal outcome. Thus, a substantial proportion of patients developed additional symptoms during their stay in the hospital. Usually, because of the publicity in newspapers, many patients prefer to get admitted for monitoring. This was clear by the observation of the 397 patients with WWS admitted during 2017–19, only 168 (42.3%) remained WWS after follow-up till discharge.

Table 4 displays the relationship of comorbidities with the age-wise distribution of patients with WWS, WS, and SD. Comorbidity was present in a minority of dengue patients (*n* = 52, 11.5%). However, the proportion of dengue cases in patients with comorbidity was higher in patients with secondary infection (37/52, 71.2%) than those with primary infection (15/52, 28.8%, *p* < 0.001). Both children with severe disease had secondary infections, and out of 12 adults with severe disease, an equal number of adults had primary and secondary infections. The only fatal case was an adult primary dengue patient. Proportion of WS cases were significantly higher in secondary dengue infections (186/276, 67.4%) than in patients with primary infections (90/177, 50.8%, *p* < 0.001).

**Table 4:** Association of age and comorbidities with severity in primary and secondary dengue infections among hospitalized dengue patients.

Category	Total	Age < 18 years	Age ≥ 18 years
Total dengue patients	453	38	415
Total primary cases	177 (39.1%)	16 (42.1%)	161 (38.8%)
WWS patients	81	9	72
With comorbidity	5 (6.2%)	0	5 (6.9%)
Without comorbidity	76 (93.8%)	9 (100%)	67 (93.1%)
*p*-value (with vs. without comorbidity)	<0.001	<0.001	<0.001
WS patients	90	7	83
With comorbidity	7 (7.8%)	0	7 (8.4%)
Without comorbidity	83 (92.2%)	7 (100%)	76 (91.6%)
*p*-value (with vs. without comorbidity)	<0.001	<0.001	<0.001
SD patients	6	0	6
With comorbidity	3 (50%)	0	3 (50%)
Without comorbidity	3 (50%)	0	3 (50%)
*p*-value (with vs. without comorbidity)	1.0	NA	1.0
Total secondary cases	276 (60.9%)	22 (57.9%)	254 (61.2%)
WWS patients	82	8	74
With comorbidity	5 (6.1%)	0	5 (6.7%)
Without comorbidity	77 (93.9%)	8 (100%)	69 (93.3%)
*p*-value (with vs. without comorbidity)	<0.001	<0.000	<0.001
WS patients	186	12	174
With comorbidity	26 (14%)	1 (8.3%)	25 (14.4%)
Without comorbidity	160 (86%)	11 (91.7%)	149 (85.6%)
*p*-value (with vs. without comorbidity)	<0.001	0.0002	<0.001
SD patients	8	2	6
With comorbidity	6 (75%)	2 (100%)	4 (66.6%)
Without comorbidity	2 (25%)	0	2 (33.3%)
*p*-value (with vs. without comorbidity)	0.13	0.32	0.56

When hospitalized (Table 4) and at first sampling (Table 3) patients with warning signs were compared, adult patients with secondary infection showed a similar pattern, i.e., a significantly higher proportion of warning signs when compared to primary infections (*p* < 0.001). As the number of hospitalized paediatric patients was low (*n* = 38), the proportion of secondary cases was comparable (22/38, 57.9%) to those with primary infections (16/38, 42.1%, *p* = 0.25). This was distinctly different from the first sampling group wherein 177/314 (51.4%) primary and 95/225 (42.2%, *p* < 0.001) secondary dengue patients reported warning signs. Further, secondary infection in the paediatric patients with warning signs was comparable in hospitalized and clinics at the time of first sampling (12/22, 54.5%, and 95/225, 42.2%, *p* = 0.37).

Table 5 describes the clinical symptoms of hospitalized severe dengue patients. Two were children (4 and 13 years of age, both secondary infections without comorbidity) while the remaining 12 were adults. Six each had primary and secondary infections. In adults, 3/6 primary and 4/6 secondary dengue patients exhibited one or multiple comorbidities. One female (46 years) with primary infection and without any comorbidity succumbed to dengue. We could identify infecting serotypes in all six primary infections and 2/8 secondary infections.

**Table 5:** Age/gender, serology, and clinical symptoms among patients with severe dengue.

Patient no.	Age / gender, infecting serotype	Serology	Symptoms
1	45/F, NA	NS1- IgM+ Secondary	Fever, chills, altered sensorium, platelet count = 3,500, liver involvement, transaminitis, SGPT = 77 IU/litre, no comorbidity
2	42/F, NA	NS1- IgM+ Secondary	Fever, muscle pain, nausea, giddiness, itching, hepatitis, platelet count = 80,000, SGPT = 1,170 IU/litre, SGOT = 3,080 IU/litre, comorbidity - diabetes, hypertension
3	27/F, NA	NS1- IgM+, Secondary	Fever, chills, ascites, anorexia, platelet count = 70,000, SGPT = 1,090 IU/litre, SGOT = 2,350 IU/litre, no comorbidity
4	25/M, DENV-1	NS1+ IgM- Primary	Fever, headache, muscle pain, nausea, WBC = 1,600, Platelet count = 37,000, SGPT = 241 IU/litre, SGOT = 1,380 IU/litre, comorbidity – TB meningitis
5	55/F, NA	NS1- IgM+ Secondary	Fever, muscle pain, joint pain, ascites, platelet count = 23,000, SGPT = 870 IU/litre, SGOT = 2,310 IU/litre, comorbidity - hypothyroidism
6	48/M, DENV-1	NS1+ IgM- Primary	Fever, headache, muscle pain, joint pain, diarrhoea, chills, seizure, WBC = 4,200, platelet count = 45,000, SGPT = 1,220 IU/litre, SGOT = 1,870 IU/litre, comorbidity - diabetes, hypertension, chronic kidney disease
7	35/M, DENV-3	NS1+ IgM+ Primary	Fever, chills, nausea, abdominal pain, muscle pain, ascites, bilateral moderate pleural effusion, borderline splenomegaly, pseudo gall bladder wall thickening, WBC = 3,500, platelet count = 15,000, SGPT = 345 IU/litre, SGOT = 1,740 IU/litre, no comorbidity
8	19/F, NA	NS1-, IgM+ Secondary	Fever, chills, nausea, muscle pain, rash, transaminitis, thrombocytopenia, mild ascites, WBC = 5,400, platelet count = 15,000, SGPT = 1,140 IU/litre, SGOT = 1,280 IU/litre, no comorbidity
9	46/F, DENV-1	NS1-, IgM+ Primary	Fever, chills, stiffness, retrosternal burning, dengue haemorrhagic shock with multi organ failure, expired, no comorbidity
10	4/F, DENV-1	NS1+, IgM+, Secondary	Fever, chills, muscle pain, lethargy, decrease in urine output, cold hypotensive shock, severe dengue, WBC = 7,500, platelet count = 19,000, no comorbidity
11	13/F, NA	NS1-, IgM+, Secondary	Fever, chills, nausea, muscle pain, hypotensive shock, severe dengue, autoimmune polyendocrinopathy syndrome type I, WBC = 2100, platelet count = 62,000, no comorbidity
12	48/M, DENV-1	NS1+, IgM+, Secondary	Fever, chills, giddiness, nausea, joint pain, muscle pain, haematuria, hematochezia malena, chronic kidney disease, petechiae, dengue haemorrhagic fever, WBC = 6,200, platelet count = 10,000, SGPT = 150 IU/litre, SGOT = 1,070 IU/litre, no comorbidity
13	39/F, DENV-2	NS1-, IgM+ Primary	Fever, chills, muscle pain, altered sensorium, hepatomegaly, platelet count = 26,000, SGPT = 990 IU/litre, SGOT = 760 IU/litre, comorbidity – cardiovascular disease
14	20/M, DENV-1	NS1+, IgM+, Primary	Fever, chills, nausea, abdominal pain, muscle pain, haematuria, melena, bleeding from gums, petechiae, no comorbidity, multi-organ failure (pseudo-thickening of gall bladder wall, mild splenomegaly, mild ascites, mild pleural effusion), WBC = 4900, platelet count = 15,000, SGPT = 350 IU/litre, SGPT = 390 IU/litre, no comorbidity

*NA-Not available.

### 3.10 Impact of the COVID-19 pandemic on the number of dengue patients during subsequent years at the Bharati hospital

Due to imposed lockdowns, the initial scare of the pandemic, high rate of hospitalisation and mortality during the Delta wave, the diagnosis and treatment for other diseases were ignored. The Bharati Hospital was a treatment centre for COVID-19. The remarkable impact on dengue was evident. As against 1,362 confirmed dengue patients in 2019 (pre-pandemic), the numbers were reduced to 127 (90.7%, 2020), 358 (74.8%, 2021), 699 (48.7%, 2022), and 676 (50.4%, 2023). However, during the 2024 dengue season, 443 dengue cases were identified till August. The cases are expected to occur till November end. It seems that the dengue activity is coming back to the pre-pandemic levels.

### 3.11 DENV serotypes circulating during 2017–19

**Figure 5:**
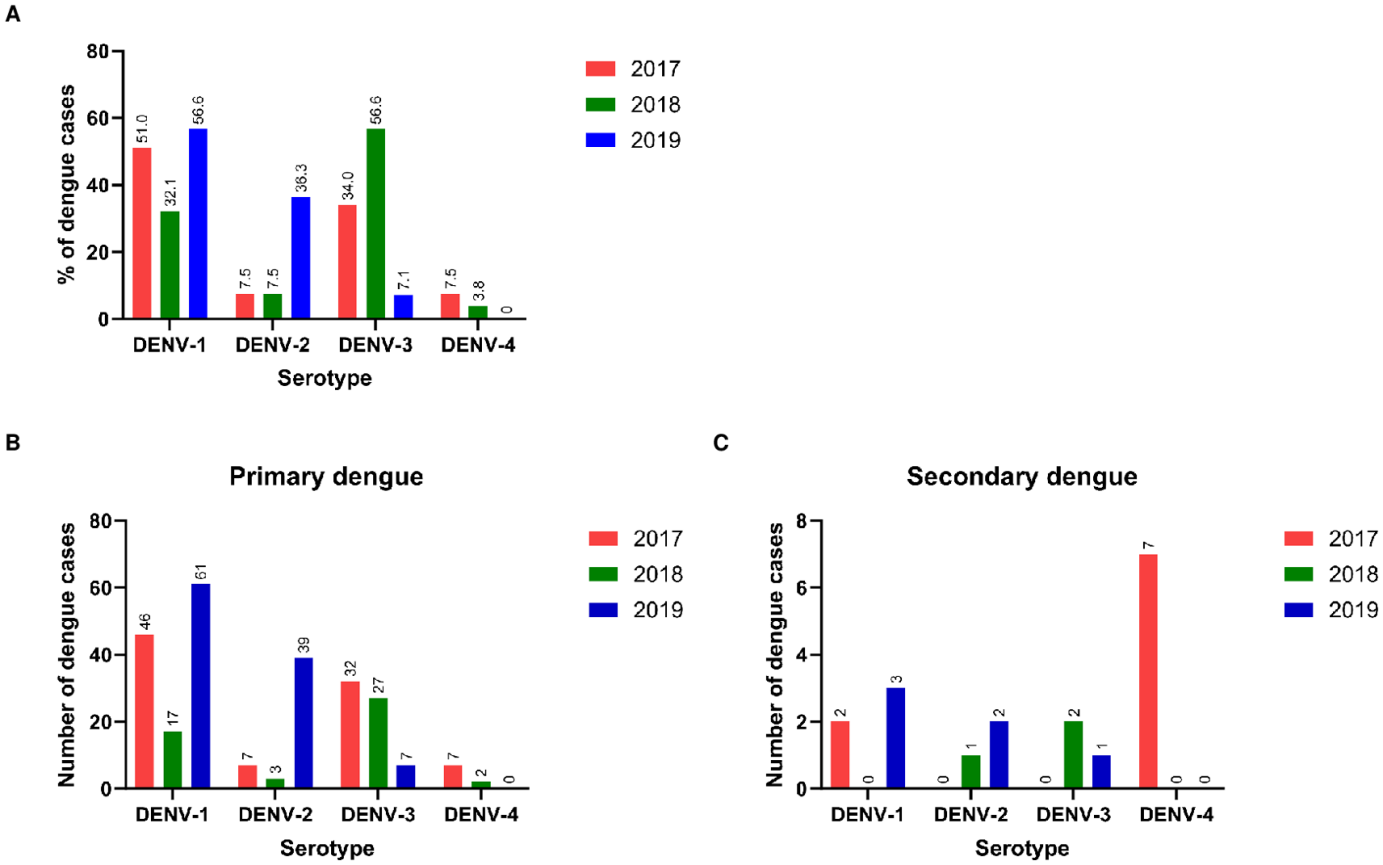
**(A)** Distribution of DENV serotypes in different years*. Year-wise serotypes detected in **(B)** primary and **(C)** secondary dengue patients. *Mixed genotypes detected: 2017 = D1 + D2 (1), 2018 = D1 + D2 (1), D2 + D3 (1), 2019 = D1 + D3 (2).

A total of 260 viral RNA-positive samples were serotyped. The pattern of DENV serotype distribution differed each year (Figure 5A). In 2017 and 2018, all 4 serotypes were detected, DENV-1 and DENV-3 being the predominant serotypes in the respective years. Serotypes DENV-2 and DENV-4 were present in a few patients. During 2019, though DENV-1 was the predominant serotype, 36.3% of patients circulated serotype DENV-2 which was infrequent during the earlier years. DENV-4 was not detected in 2019. We could not compare serotypes responsible for primary/secondary infections as NS1 positivity and consequent viral RNA detection were low in the secondary dengue patients (18/260, 6.9%). Interestingly, the DENV-4 serotype was detected in both primary (*n* = 7) and secondary (*n* = 7) dengue patients studied in 2017 (Figures 5B,C). Serotypes among patients with or without warning signs in a season were not different. Of the 14 patients with severe disease, serotyping was available for 8 patients (6/6, primary and 2/8, secondary dengue). Irrespective of the type of infection, all circulated the serotype predominant in that year.

## 4 Discussion

A comprehensive analysis of the association of dengue disease and the virus with primary/secondary infections and serostatus studied for three consecutive years in one of the 15 administrative wards of a metropolitan city is presented here. All the patients from 10 private clinics situated in different parts of the ward, one corporation hospital charging minimum charges, and a private, tertiary care hospital with 800 beds were included. Additionally, we provide significant information based on a retrospective analysis of 453 dengue patients admitted to the tertiary care hospital (Bharati hospital).

As dengue diagnosis is impacted by the type of kits used, we used previously validated ELISA kits for NS1 and IgM detection (21). Therefore, our data provides an accurate picture of the ward during 2017–19. The study underscores a need to conduct both NS1 and IgM tests for an accurate dengue diagnosis. If only NS1 or IgM were used for the present study, 25.5% or 43% of the total dengue patients, respectively, could have been missed leading to substantial underreporting of the disease. Importantly, >90% of the primary dengue patients were positive for NS1, but IgM alone positivity was dominant among secondary infections in adults. Clearly, the use of a single marker is not advisable.

Another important issue is the lack of adequate testing. Though Pune Municipal Corporation and private hospitals/labs provide dengue diagnosis, the number of patients seeking dengue diagnosis remains low, especially when the disease is mild. This is reflected in the differences in the figures of yearly dengue cases for the entire Pune city as reported by PMC and our study based on 10 private clinics, two major hospitals, and a paediatric hospital (for 2 years) of a single ward: 1,691 (910), 682 (830), and 1,407 (1,274) (PMC website; yearly information). Given the large number of laboratories throughout the city providing diagnosis, there seems to be a lack of communication among the clinics and PMC or the use of a single diagnostic marker by a substantial number of laboratories. Using kits (rapid and ELISA) with different performance characteristics could probably be one of the factors.

Secondary infections are likely to progress to severe disease and, several factors such as age, the time gap between the first and second DENV infections, and serotype influence the severity of the second heterotypic dengue infection. As Pune has been documented to be hyperendemic for dengue (8), we did evaluate secondary infections as judged by the presence of high-tired IgG-anti-DENV antibodies using IgG-capture ELISA from Panbio. Our study estimates (1) disease burden in an administrative ward by diagnosing the patients visiting private clinics, OPD of a corporation, and tertiary care hospitals and dengue patients on admission at the tertiary care hospital (2) disease severity in the patients hospitalized in the tertiary care hospital by retrospective analysis of hospital records. Considering the first estimate, the gross underestimation of yearly dengue patients reported to the Pune Municipal Corporation was obvious. This part of the study revealed that irrespective of age, a substantial proportion of primary infections progress to severe disease while the secondary infections remain mild. It is pertinent to note here that of the 14 patients with severe disease, 6 had primary, and 8, including 2 children had secondary dengue infections emphasizing a substantial contribution of primary dengue in causing severity. Whether clinic or hospital-based, secondary infection was predominant in adults with warning signs (58% or 69% respectively). Additionally, almost 50% of the primary dengue patients did report warning signs. It, therefore, appears that ADE-dependent and -independent mechanisms are operative in causing severity. The possibility of high-tired antibodies due to exposure to multiple serotypes protecting some adults from severe disease cannot be ruled out. Evaluation of non-ADE-dependent mechanisms needs immediate evaluation.

Studies from Southeast Asian countries have shown secondary infections to be primarily responsible for severity in children (22–24). An elegant study from Thailand reported that only 4/59 dengue cases in children had primary infection and presented as mild disease whereas 49% of the remaining patients with secondary dengue infections developed DHF (25). Analysis of data (1994–2006) from Bangkok, Thailand showed a strong association of secondary disease with DHF/more severe DHF (26). In contrast, in a retrospective study from Brazil (2000–2014), H/O previous dengue did not emerge as an independent variable for severity and hospitalization (27). In Kenya, despite repeated outbreaks, the clinical presentation was predominantly mild suggesting differences from the Southeast Asian countries (28). Studies conducted in 2015–16 and 2019–20 at a children’s hospital in Argentina showed that 61% of cases were mild, and 33 and 6%, respectively, developed warning signs / severe disease (29). Secondary infections were not diagnosed. In-depth research is needed to understand the differential outcomes of secondary infections noted in the present study. We showed that underlying comorbidities did not influence the outcome of dengue infection. Genetic factors seem to be influencing disease outcomes in secondary infections. The role of complement receptor type 1 and 2 (CR1 and CR2) gene polymorphisms, plasma protein levels (30), and interferon-gamma gene diplotype (AA-rs2069716 / AG-rs2069727) in disease severity is reported (31). Genetic variation in DENV-2 and -3 have been associated with the disease outcome (32–35).

To understand circulating serotypes in general and those present in secondary infection and as heterotypic infection is responsible for secondary infection and severity, we managed to serotype DENV from 260 patients. Unfortunately, a small proportion of patients with secondary infections could be serotyped. Antibody-based serotyping would be the method of choice in secondary dengue patients as majority of them were viremia-negative. Two observations are noteworthy. Firstly, the uncommon serotype DENV-4 was present in primary (*n* = 7) and secondary (*n* = 7) infections to the same extent. Secondly, out of 14 severe disease patients, serotypes were identified in 8 severe disease patients including 2/8 secondary and 6/6 primary infections. Taken together, our data is inconclusive in understanding the role of heterotypic infections in disease severity. Future studies should be specifically designed to address this important issue.

Comparison of median ages in primary and secondary infections among the patients with or without warning signs led to some interesting findings. In both patient categories, the median age was higher in secondary infections. However, WS patients were younger than the WWS category. Here, we would like to point out a long-term, elegant study from Thailand (36). During 1981 and 2017, the mean age of DHF cases increased from 8.1 to 24.3 years. Demographic transition was concluded to be the driving force for the observed change and shifting of DHF towards individuals with comorbidities was suggested. So far, comorbidity has not influenced disease severity in Pune, India. Our study classifying patients based on WHO-2009 spanned over only 3 years and should continue to observe changing trends, if any. Of note, we did not come across many severe disease cases.

Globally and from India, symptoms of dengue patients presenting with different clinical manifestations have been reported (1, 2, 12, 37–41). Our study is restricted to symptoms alone and except for severe disease patients, details of clinical laboratory tests were not available. Some of the symptoms differentiating dengue and non-dengue cases in the present study were comparable to cases in Kenya in 2016–17, though certain differences did exist (28). One of the significant findings of our study is providing age-wise comparative data on symptoms presented by WS and WWS patients with primary or secondary infections, at the time of first sampling. In our patient series, involvement of the liver was common among paediatric (abdominal pain) and adult (nausea/vomiting) secondary dengue infections. An age-dependent rise in the symptoms during primary infections is a noteworthy observation. In general, children are known to develop a substantial proportion of subclinical infections than adults. Like other reports, we did find that patients during the viremic phase were more symptomatic. Based on the data on 5642 dengue patients, adverse outcome was associated with higher viremia irrespective of other important parameters influencing severity (23). The retrospective analysis of 453 hospitalized patients yielded much-needed information emphasizing a need to undertake OPD and hospital-based programs simultaneously that will complement each other. Any single format will miss some precious information.

India is a large country with varied geographic, ecologic, and cultural diversity, and region-specific differences in the epidemiology of disease are expected. To understand the differences in serotype distribution and disease presentations between Pune (western India) and the other parts of the country, we compared recent reports describing serotypic variations for ≥2 years. During the 3 years of our study, serotype prevalence varied from predominance of DENV-1 in 2017 and 2019 and, DENV-3 in 2018. As against all 4 serotypes in 2017 and 2018, DENV-4 was absent in 2019 with a higher proportion of DENV-2 when compared to the previous years. In Kerala (south India), DENV-1 was predominant in 2017 (DENV-2 and DENV-3 present) while in 2018 and 2019, DENV-2 was most prevalent. Only in 2019, all 4 serotypes were present (9). In another state from south India, all 4 serotypes were detected in 2017, DENV-4 being the most predominant (10). In central India, both in 2019 and 2021, all 4 serotypes were detected, DENV-2 being predominant (11). A study from Uttar Pradesh (north India) documented DENV-3 to be the predominant serotype during 2018 and 2019 (DENV-2 and DENV-1 detected) whereas during 2020 and 2021, the DENV-2 serotype was predominant. DENV-4 was detected only in 2019 and 2021 wherein all 4 serotypes were circulating (12). Another study from eastern UP detected all 4 serotypes during 2018–19, DENV-2 being the most prevalent (13). Serotypic analysis during 2011–17 at Delhi showed DENV-2 as the predominant serotype during 2011–2015 that was replaced by DENV-3 during 2016–2017 (14). Clearly, despite the differences seen in the predominant serotypes in a year, detection of all 4 serotypes was noted in all parts of the country, though not every year. A Pan-India study conducted by the Indian Council of Medical Research in 2018 concluded that the predominance of a serotype was not geographically restricted but varied in different states from the same part of India (42). Hyperendemicity of dengue and exposure to multiple virus serotypes may be responsible for the reduced severity of secondary dengue in India.

Though limited to a single tertiary care hospital in Pune, we observed a remarkable reduction of 90.7% in dengue cases in 2020 compared to the pre-pandemic year 2019. Though reduced, a gradual increase in dengue cases was seen from 2021 to 2023. It seems that the virus has bounced back in 2024. In the state of Maharashtra wherein Pune city is situated, as compared to 2019 (*n* = 14907) the numbers reduced by 77.5% (2020), 14.7% (2021), and 42.5% (2022) reaching 19,034 in 2023 (43). During the 2024 ongoing dengue season, a higher number is being reported. Our data from the western Indian state of Maharashtra agrees with year-round mosquito surveillance data from a southern Indian state. In contrast to 42.6% DENV positivity in 2019, during the subsequent years (2020-April 2024) a drastic reduction to 3–8% was recorded (44).

A statistical modelling study encompassing 23 countries from southeast Asia and Latin America throughout 2020 revealed that various measures enforced to combat the COVID-19 pandemic were consistently associated with reduced dengue transmission (45). Importantly, these were not related to underreporting or seasonal/extra-seasonal cycles. The number of cases declined dramatically in 2020 (90,304) and 2021 (26,365) compared to the year 2019 (130,101), which coincided with the COVID-19 pandemic. In Malaysia, the dramatic decline in dengue cases in 2020 and 2021 coincided with the lockdowns and subsequently, the disease reverted to pre-pandemic levels (46). Taken together, these studies emphasize the role of human movement in dengue transmission.

Our study has certain limitations. Firstly, the study is restricted to a single administrative ward of the city. Secondly, we did not collect follow-up samples. Despite these limitations, we provide a comprehensive picture of dengue disease concerning age, primary/secondary infections, disease duration, serotypes, and hospitalization. These findings are indeed important for dengue vaccine efficacy studies. In most countries including India with a high dengue burden and failure of the mosquito control programs, a vaccine with high efficacy would be welcome.

## Data Availability

The original contributions presented in the study are included in the article/supplementary material, further inquiries can be directed to the corresponding author.

## Footnotes

^1^https://ncvbdc.mohfw.gov.in/index4.php?lang=1&level=0&linkid=431&lid=3715

^2^https://www.census2011.co.in/census/district/359-pune.html

^3^www.ncbi.nlm.nih.gov/BLAST
